# Ninein is essential for the maintenance of the cortical progenitor character by anchoring the centrosome to microtubules

**DOI:** 10.1242/bio.20135231

**Published:** 2013-06-10

**Authors:** Hiroshi Shinohara, Nobuyuki Sakayori, Masanori Takahashi, Noriko Osumi

**Affiliations:** Division of Developmental Neuroscience, United Core Centers for Advanced Research and Translational Medicine (ART), Tohoku University Graduate School of Medicine, Sendai, Miyagi 980-8575, Japan; * Present address: Department of Histology and Neuroanatomy, Tokyo Medical University, 6-1-1 Shinjuku, Shinjuku-ku, Tokyo 160-8402, Japan; ‡ Present address: Division of Biology, Center for Molecular Medicine, Jichi Medical University, Shimotsuke, Tochigi 329-0498, Japan

**Keywords:** Interkinetic nuclear migration, Cortical progenitor cells, Mictrotubules, Centrosome, Pax6, Ninein, Rat

## Abstract

The mammalian cerebral cortex develops from proliferative apical progenitor cells (APs) that exhibit cell cycle-dependent nuclear movement (interkinetic nuclear migration; INM), which may be important for efficient and continuous production of neurons. The Pax6 transcription factor plays a major role in INM by regulating various downstream molecules. We have previously observed abnormal INM and unstable localization of the centrosome in APs of the *Pax6* homozygous mutant rat embryo. To understand the mechanisms of INM, we focused on the centrosomes of APs. One of the centrosomal proteins, ninein, is specifically localized in the centrosome of APs. We observed a dramatic downregulation of ninein in APs of the *Pax6* mutant. Moreover, knockdown of *ninein* by RNAi induced ectopic distribution of reduced numbers of BrdU-positive (S-phase) and PH3-positive (M-phase) cells. Furthermore, time-lapsed imaging demonstrated that knockdown of ninein *in vivo* induced abnormal INM. Finally, we observed impaired microtubule regrowth in neural progenitors taken from *Pax6* homozygous mutant rat embryos, which was recovered by via *ninein* overexpression. We also found that *ninein* knockdown enlarged the surface size area of apical endfeet of the APs. Our results suggest that ninein plays a role in the molecular machinery essential for INM by connecting microtubules to the centrosome.

## Introduction

The cortical primordium consists of apical progenitor cells (APs) that are highly polarized, with long apical and basal processes. APs form an adherence junction complex with the centrosome, which is located towards their most apical side. The nuclei of APs migrate in two dimensions along the apical–basal axis during the cell cycle. This phenomenon is termed interkinetic nuclear migration (INM) and is considered important for the efficient and continuous production of neurons. Early work indicates that microtubules are essential for INM (Messier and Auclair, 1973), and several cytoskeleton-related factors (e.g. actin, myosin II, actomyosin, Lis1, kinesin3, dynein, dynactin, Tpx2, Rac, and laminin) have also been implicated in INM (Tsai et al., 2005; Del Bene et al., 2008; Minobe et al., 2009; Norden et al., 2009; Schenk et al., 2009; Tsai et al., 2010; Tsuda et al., 2010; Kosodo et al., 2011; Spear and Erickson, 2012). Moreover, centrosomal proteins, such as Cep120, TACC and Hook3, which are involved in the regulation of microtubules, can also regulate INM (Xie et al., 2007; Ge et al., 2010), thus demonstrating a more complex molecular mechanism underlying INM than originally was thought.

Pax6 is a transcription factor that is essential for embryonic and adult neurogenesis (Götz and Huttner, 2005; Manuel and Price, 2005; Hevner et al., 2006; Osumi et al., 2008). We have previously reported abnormal INM phenotypes in the neocortex of the *Pax6* homozygous mutant rat (*rSey^2^/rSey^2^*), including unsteady ascent and descent movement, ectopic cell division in the basal side of the ventricular zone (VZ), and unstable centrosomal positioning during the S to M phase of the cell cycle (Tamai et al., 2007; Osumi et al., 2008). Because Pax6 can regulate the expression of various downstream genes (Holm et al., 2007; Numayama-Tsuruta et al., 2010), we hypothesized that centrosomal instability during INM may be due to dysregulation of certain centrosomal molecule(s) in *Pax6*-deficient conditions.

## Materials and Methods

### Animals

Pregnant Sprague–Dawley (SD) rats were purchased from Japan Charles River (Yokohama, Japan). The *Pax6* heterozygous mutants (*rSey^2^/+*) of SD background rats (Osumi et al., 1997) were intercrossed in our laboratory to obtain homozygous *rSey^2^/rSey^2^* embryos. These embryos were distinguished from the wild-type and heterozygous littermates by an eyeless phenotype. The midday of the vaginal plug was designated as embryonic day 0.5 (E0.5). All animal experiments were carried out in accordance with the National Institute of Health Guidelines and the Guide for the Care and Use of Laboratory Animals. The Committee for Animal Experimentation of Tohoku University Graduate School of Medicine and the Tokyo Medical University Animal Committee approved the experimental procedures.

### Immunohistochemistry

Immunohistochemical straining was performed as previously described with slight modifications (Osumi et al., 1997). On E17.5 (which corresponded to E15.5 in the mouse), wild-type and *rSey^2^/rSey^2^* rat embryos were transcardially perfused with 4% paraformaldehyde (PFA) in PBS (phosphate buffered saline, pH 7.4), and the cortex primordium was resected and fixed a second time in 4% PFA for 20 minutes at 25°C. The neocortex was sectioned with a cryostat (CM3050, Leica, Heerbrugg, Switzerland), and the sections were immersed in 2% goat serum in TBST (Tris-buffered saline plus 1% Triton X-100, Sigma, St. Louis, MO) for 1 hour. Cortical sections were then incubated with primary antibodies at 4°C overnight and then washed in TBST, followed by incubation with the appropriate fluorescence-conjugated secondary antibodies and DAPI (Sigma) at room temperature (RT) for 1 hour. The primary antibodies used were as follows: anti-Pax6 (1:1000, rabbit polyclonal) (Inoue et al., 2000), anti-phospho histone H3 (1:200, rabbit polyclonal, Upstate Biotechnology, Billerica, MA), mouse anti-γ-tubulin (1:200, clone GTU-88, Sigma), anti-ninein (1:400, rabbit polyclonal, kindly provided by Dr K. Hayashi) (Ohama and Hayashi, 2009), anti-pericentrin (1:500, rabbit polyclonal, Covance, Berkeley, CA), mouse anti-active-caspase3 (1:100, clone C92-605; Becton Dickinson, Franklin Lakes, NJ), mouse anti-BrdU (1:200, clone B44, Becton Dickinson), rat anti-BrdU (1:200, AbD Serotec, Kidlington, UK), anti-Rat Ki67 (1:25, clone MIB-5, Dako, Glostrup, Denmark), anti-Tbr2 (1:1000, rabbit polyclonal; Chemicon, Billerica, MA), chicken anti-GFP (1:1000, Abcam, Cambridge, MA), mouse anti-N-Cadherin (1:2000, clone 32/N-Cadherin; Becton Dickinson), mouse anti-Neurogenin2 (1:20, clone 7G4, kindly provided by Dr D. Anderson) (Lo et al., 2002) and mouse anti-ZO-1 (1:5, clone T8-754, kindly provided by Dr S. Tsukita) (Itoh et al., 1993). Alexa Fluor 405-conjugated anti-mouse IgG, Alexa Fluor 488-conjugated anti-rabbit or anti-chicken IgG, Alexa Fluor 555-conjugated anti-mouse or anti-rabbit IgG goat antibodies (Invitrogen, Carlsbad, CA), and Cy5-conjugated anti-rat IgG donkey antibody (Jackson ImmunoResearch Laboratories, West Grove, PA) were used as secondary antibodies. The sections were examined under a fluorescent microscope (Axioplan 2, Carl Zeiss, Jena, Germany), and confocal images were acquired using a confocal microscope (LSM5 PASCAL or LSM510 META, Carl Zeiss). The endfoot area of GFP-expressing cells was measured randomly using at least 4 GFP-transfected telencephalic hemispheres, as previously described (Nishizawa et al., 2007).

### Real-time quantitative PCR

Real-time quantitative PCR was performed using a Light Cycler instrument according to the protocol recommended by the manufacturer (Roche Diagnostics, Indianapolis, IN). Template double-stranded DNA was synthesized from 2.5 µg of cDNA prepared from both the wild-type and *rSey^2^/rSey^2^* samples. Real-time quantitative PCR was performed after optimizing the MgCl_2_ concentration using the following primers: *ninein* (5′-GGAGGAGCTTGACAACAGGA-3′ and 5′-ATGTCTGTGTACGGTGTGCG-3′); *pericentrin* (5′-ATCAAATGAGAAGGCGGTGT-3′ and 5′-GCTTTTGTGAGGGCATCGTT-3′); and *γ-tubulin* (5′-GGGACCCTCATCTGCCTTAC-3′ and 5′-CCCCTAAGCCAACAGACAGT-3′) (Nihon Gene Research Laboratories, Miyagi, Japan). Detailed conditions of the real-time PCR reaction can be supplied upon request.

### Expression plasmids

We labeled APs with *pCAX-EGFP* and *pCAX-EGFP-NLS* as previously described (Tsunekawa et al., 2012). *pCAGGS-mRFP* was provided by Dr M. Uchikawa (Campbell et al., 2002). *pEGFP-ninein* and *GFP/Nter-ninein* plasmids were provided by Dr M. Bornens (Bouckson-Castaing et al., 1996; Delgehyr et al., 2005).

### RNAi

RNA interference (RNAi) was performed using Stealth RNAi, which is a novel, modified siRNA with enhanced stability (Invitrogen, Carlsbad, CA). Two 25-mer Stealth RNAi duplexes targeting *ninein* and one 25-mer scramble control Stealth RNAi duplexes were obtained using the BLOCK-iT RNAi design program (Invitrogen); the sequences were as follows: *ninein* 947si duplexes (5′-UUUAGUUGUCUAUAGGGAGUAGAUG-3′ and 5′-CAUCUACUCCCUAUAGACAACUAAA-3′); *ninein* 2580si duplexes (5′-AUUACACUCUGUUUCCAUUUCUUCC-3′ and 5′-GGAAGAAAUGGAAACAGAGUGUAAU-3′); and scrambled control siRNA duplexes for *ninein* (5′-AUUUCAACUCUCUGUUCCUAUUUCC-3′ and 5′-GGAAAUAGGAACAGAGAGUUGAAAU-3′). Note that the target site of *ninein* 947si is designed for the rat *ninein* sequence and does not recognize the mouse sequence used for overexpression.

### *In utero* electroporation

*In utero* electroporation of rat embryos was performed as previously described (Takahashi et al., 2002), with modifications. Pregnant wild-type rats at E15.5 or E16.5 and *rSey^2^/+* rats at E15.5 (for rescue experiments) were anesthetized with 97% 2,2,2-Tribromoethanol in *tert*-amyl alcohol. After cleaning the abdomen with 70% ethanol, a midline laparotomy of approximately 3 cm was performed, and the uterus was removed. Stealth RNAi for ninein or control and *pCAX-EGFP* were diluted with PBS (final RNAi concentration 200 µM), and 1–3 µl of the solution was injected into the lateral ventricle with a mouth-suction glass capillary pipette. Square pulses (50 V, 50 milliseconds, 5 times at 1 second intervals) were delivered to the neocortex with tweezer-type electrodes, which consisted of a pair of round platinum plates that were 3 mm in diameter (LF650P3, BEX, Tokyo, Japan), and an electroporator (CUY21, BEX). Electroporated embryos were fixed at E17.5. To analyze the distribution of S-phase cells, neuroepithelial cells electroporated at E15.5 were labeled with BrdU by intraperitoneal injection of PBS solution (140 mg/kg) for 15 minutes prior to fixation. To measure the labeling index of the cell cycle exit, embryos were pulse-labeled with BrdU for 24 hours (at E16.5). The cell cycle exit rate was calculated as the ratio of Ki67-negative, BrdU-positive cells to all BrdU-positive cells, which represents the fraction of cells that become post-mitotic among the daughters of the S-phase cells during the 24-hour period following E17.5.

### Time-lapsed imaging

The procedures were performed as previously described (Tamai et al., 2007), with modifications. Brains were dissected out of E17.5 embryos, and the pia mater was removed. The isolated brains were transferred to a DMEM/F12 medium. The neocortex was electroporated with *pCAX-EGFP-NLS* and *pCAGGS-mRFP* to visualize the nuclei and the radial glial cell morphology, respectively. The labeled neocortex was manually cut into slices, which were then embedded in collagen gel (Nitta Gelatin, Osaka, Japan). The behavior of the labeled cells was observed under an inverted fluorescent microscope (Axiovert 200M, Carl Zeiss) and recorded by confocal microscopy (LSM510 Meta, Carl Zeiss) using a 20× objective lens (Plan-Apochromat, NA = 0.75, Carl Zeiss). Slices were cultured on a small incubator chamber (Tokken, Chiba, Japan) equipped on the stage of the inverted microscope. Gas consisting of 5% CO_2_/20% O_2_ was used and controlled by a gas mixture control (TK-MIGM01, Tokken). EGFP-NLS and mRFP were sequentially excited with a 488 nm Ar laser and a 543 nm HeNe laser at the low power setting. Three µm Z-stack images were captured automatically at 30 minute intervals for 24 hours, and movies were constructed by merging the Z-stack images (510Meta software). The amount of distance traveled by nuclei was manually measured at 30 minute intervals. The velocities of nuclear movements were calculated as previously described (Ge et al., 2010).

### Neural stem cell culture

The generation and differentiation of undifferentiated proliferating cells of the embryonic forebrain were performed as previously described (Reynolds et al., 1992), with minor modifications. Briefly, the neocortex was dissected from rat embryos at E17.5, collected in Tyrode's solution, and then transferred to the medium hormone mixture (MHM) composed of DMEM-F12 (1:1) (Invitrogen), 0.6% glucose, HEPES buffer (5 mM), insulin (25 µg/ml) (Wako Pure Chemical Industries, Osaka, Japan), transferrin (100 µg/ml) (Sigma), progesterone (20 nM) (Sigma), putrescine (60 µM) (Sigma) and selenite (30 nM) (Sigma). Tissue pieces were mechanically dissociated with a micropipette and then passed through 40-µm nylon mesh (Becton Dickinson). Cells were seeded at 2.0×10^5^ cells/ml into the MHM, which also contained 20 ng/ml EGF (PEPROTECH, Rocky Hill, NJ) and 10 ng/ml bFGF (PEPROTECH) together with 2 µg/ml heparin (Sigma), and were maintained in a humidified incubator at 37°C with 95% atmospheric air and 5% CO_2_. Fresh media containing 20 ng/ml EGF, 10 ng/ml bFGF and 2 µg/ml heparin were added every other day. Cells were cultured for 5–7 days *in vitro* (DIV) to form neurospheres. The neurospheres were enzymatically dissociated and plated onto poly-L-ornithine- and laminin-coated dishes at a density of 3.5×10^4^ cells/cm^2^ with EGF, bFGF and heparin and then processed further for drug experiments, transfections and immunocytochemistry. *pEGFP-ninein* and *pEGFP-C1* (Clontech) plasmids were transfected into cells using Lipofectamine 2000 (Invitrogen), and microtubule regrowth assays were performed after 24 hours.

For immunocytochemistry, cells were fixed in 4% PFA for 10 minutes at RT. After being rinsed with PBS, the cells were immersed in 3% bovine serum albumin in PBST (0.3% Triton X-100) for 1 hour at RT and then incubated with α-tubulin antibody (1:2000, Sigma) overnight at 4°C. After being rinsed with PBS, the cells were incubated with Alexa Fluor 555-conjugated anti-mouse IgG goat antibody (Invitrogen) for 1 hour at RT, and the cell nuclei were counterstained with DAPI. Cell images were captured by confocal microscopy (LSM5 PASCAL, Carl Zeiss).

### Microtubule regrowth assay

Using cultured APs, microtubule regrowth assays were performed as previously described (Delgehyr et al., 2005; Krauss et al., 2008), with modifications. Microtubules were depolymerized in 33 µM nocodazole (Calbiochem, Darmstadt, Germany) in MHM for 30 minutes at 37°C, and then cultured APs isolated from the wild-type and *rSey^2^/rSey^2^* rat embryos were washed with MHM and incubated at 37°C to allow for regrowth. The cells were fixed at various time intervals in 4% PFA for immunocytochemistry as described above, and quantifications were performed using three repetitions.

### Statistical analyses

The data are reported as the mean±s.d.; analyses for statistical significance were conducted using Student's *t*-test.

## Results

### The centrosomal protein ninein is downregulated in the *Pax6* mutant rat

**Fig. 1.**
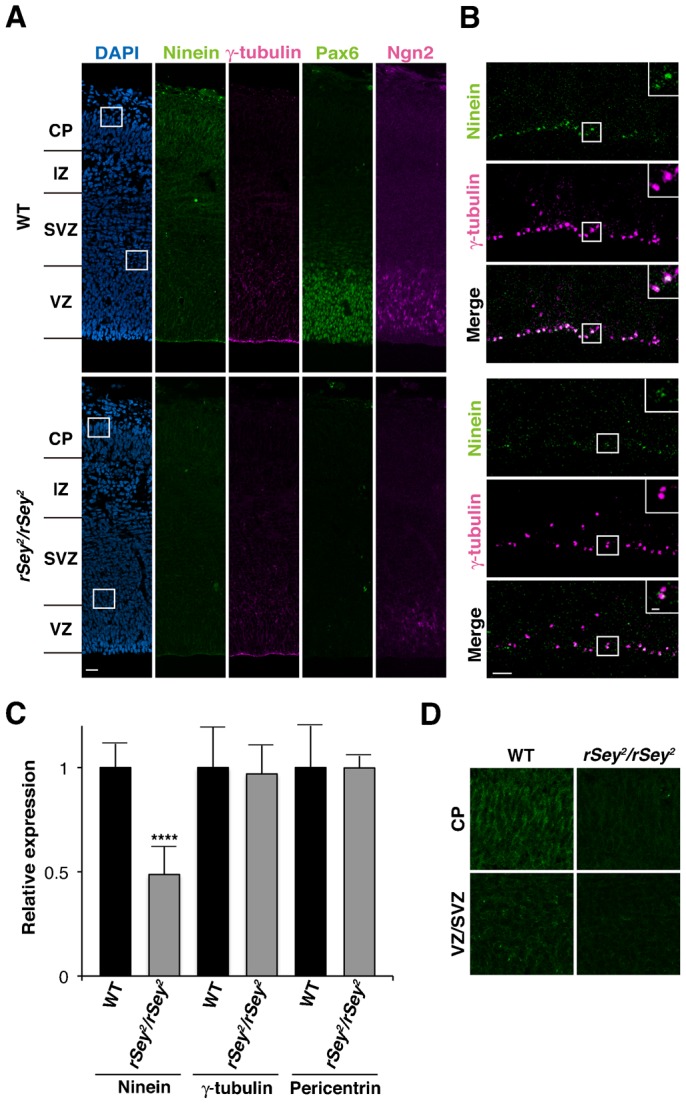
Ninein localizes at the centrosome of apical progenitor cells and is downregulated in the *Pax6* mutant rat. (**A**) Immunoreactivity of the two centrosomal protein, ninein (green) and γ-tubulin (magenta), Pax6 (green), and Ngn2 (magenta) in the neocortex of E17.5 wild-type (WT) (upper panels) and *rSey2/rSey2* (lower panels) embryos. Cell nuclei are counterstained with DAPI (blue). (**B**) Higher magnification of the apical region of E17.5 WT (upper panels) and *rSey2/rSey2* (lower panels). Note that the expression of ninein is co-localized on the centrosome in the WT (insets, three upper panels), but centrosomal localization of ninein is impaired in *rSey2/rSey2* (inset, three lower panels). (**C**) Relative expression levels of *ninein*, *γ-tubulin*, and *pericentrin* transcripts in the WT (black) and *rSey2/rSey2* (gray) neocortex were determined using real-time quantitative PCR (*n* = 12, ****P<0.0005). (**D**) Immunoreactivity of the ninein in the CP and VZ/SVZ region of Fig. 1A. VZ, ventricular zone; SVZ, subventricular zone; IZ, intermediate zone; CP; cortical plate. Scale bars: 20 µm in A, 5 µm in B, 1 µm in an inset of B.

We focused our investigation on ninein, which is reported to localize to the centrosome in mouse APs (Ohama and Hayashi, 2009; Wang et al., 2009). Using immunostaining, we observed the specific localizations of ninein in the centrosome of APs; the localization of neither intermediate progenitors (IPs) nor neurons was observed at E17.5 in the rat neocortex (Fig. 1A,B, upper panels). Interestingly, the expression of ninein was dramatically downregulated in the developing neocortex of *rSey^2^/rSey^2^*, although the expression of other centrosomal proteins, γ-tubulin (Fig. 1A,B, lower panels) and pericentrin (data now shown),was unchanged. This result was confirmed at the mRNA level; the expression of ninein, but not that of γ-tubulin or pericentrin, was reduced in *rSey^2^/rSey^2^* (gray bars in Fig. 1C) compared with the wild-type (black bars in Fig. 1C). The expression of ninein was also downregulated in the cortical plate region and the subventricular zone (SVZ) (Fig. 1D). These data suggest that Pax6 regulates the centrosomal protein ninein at the transcriptional level in APs.

### Ectopic localization of APs in the *ninein* knockdown neocortex

**Fig. 2.**
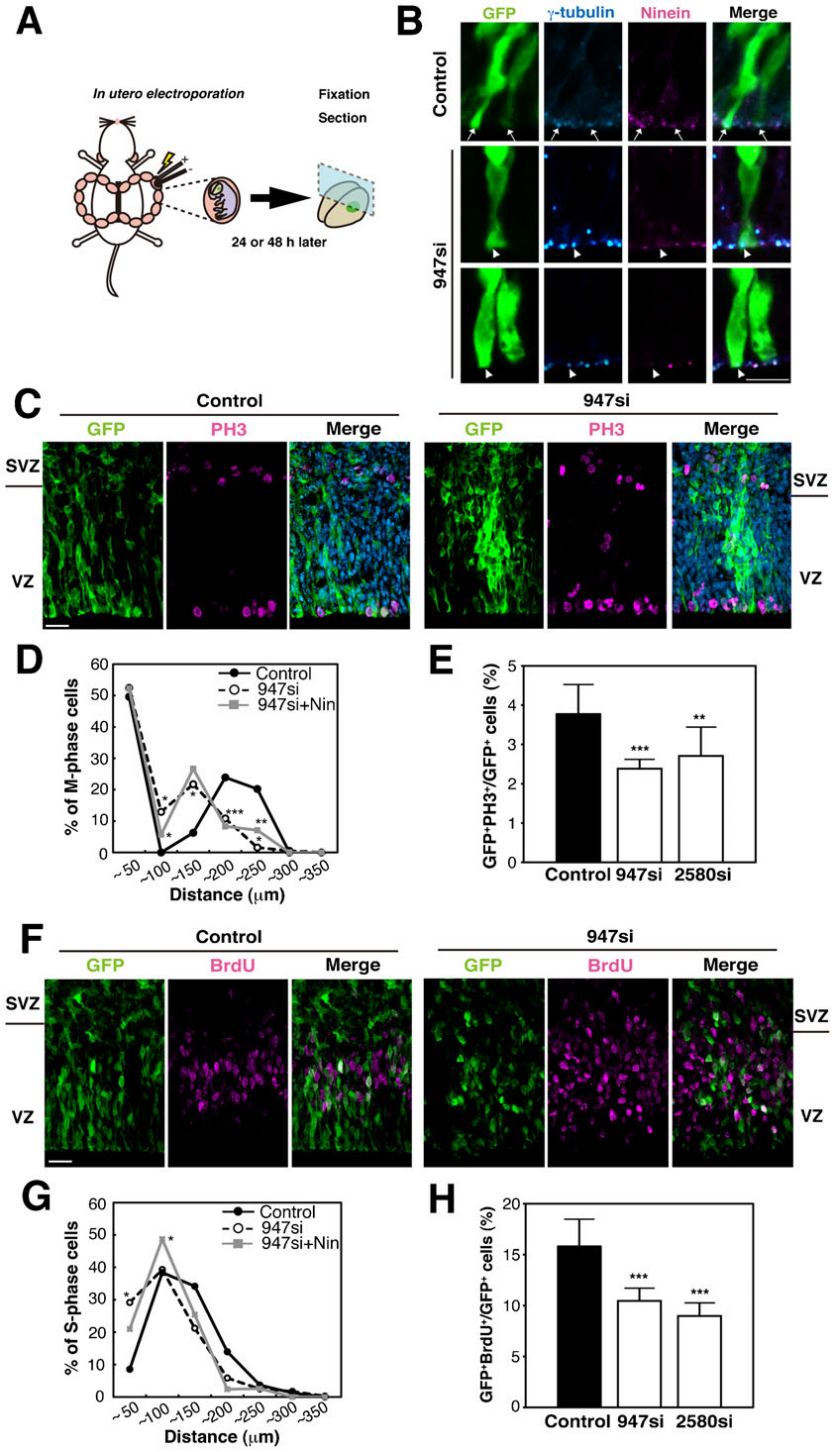
The loss of ninein function randomizes the distribution of S- and M-phase cells. (**A**) Schematic representation of a knockdown experimental procedure by *in utero* electroporation; siRNAs together with a GFP expression vector were transfected into the E15.5 rat neocortex, and the brains were dissected out 24 or 48 hours after electroporation (corresponding to E16.5 or E17.5, respectively). (**B**) Immunoreactivity of GFP (green), γ-tubulin (blue) and ninein (magenta) together with a merged image in control (upper panels) and *ninein* siRNA (947 si)-transfected (lower panels) cells. Arrows indicate the position of the centrosome of GFP-labeled neuroepithelial cells. It is of note that ninein expression is knocked down in GFP^+^ cells (arrowheads). (**C**) Immunoreactivity of GFP (green) and PH3 (magenta) in control (left) and *ninein* siRNA-transfected (right) cells. (**D**) Graphs showing the distribution of M-phase cells in *ninein* siRNA transfected (solid line), control (dotted line) and rescued (947 si plus *pEGFP-ninein*, gray line) cells. The number of PH3^+^GFP^+^ cells was calculated at 50 µm intervals from the apical surface up to 350 µm away to show a percentage of the labeled cells in each area against the total PH3^+^GFP^+^ cells (*n* = 5–6, *P<0.05, **P<0.01, ***P<0.005). (**E**) The mitotic index of *ninein* knockdown cells is reduced as shown by the percentage of GFP^+^PH3^+^ cells in the ventricular zone (VZ) (*n* = 7–18, **P<0.01, ***P<0.005). (**F**) Immunoreactivity of GFP (green) and BrdU (magenta) in control (left) and *ninein* siRNA transfected (right) cells. (**G**) Graphs showing the distribution of S-phase cells in *ninein* siRNA transfected (solid line), control (dotted line) and rescued (gray line) cells. Neuroepithelial cells in the dorsal telencephalon were electroporated at E15.5, cultured for 48 hours, and pulse-labeled with BrdU for 15 minutes before sampling. The number of BrdU^+^GFP^+^ cells was calculated 50 µm intervals from the apical surface up to 350 µm away to show a percentage of the labeled cells in each area against total BrdU^+^GFP^+^cells (*n* = 3, *P<0.05). (**H**) BrdU incorporation is reduced in the *ninein* knockdown neocortex as shown by the percentage of BrdU^+^GFP^+^ cells against total GFP^+^ cells in the VZ (*n* = 3, ***P<0.005). VZ, ventricular zone; SVZ, subventricular zone. Scale bars: 5 µm in B, 20 µm in C,F.

To determine the role of ninein in INM, we performed functional knockdown experiments using small interference RNA (siRNA) against *ninein* mRNA. Samples of E15.5 rat neocortex were co-electroporated with *ninein* siRNA (947 si or 2580 si) or with a scrambled control siRNA paired with a GFP expression vector; the electroporated rat brain tissue was dissected out at E17.5 (Fig. 2A). As expected, the expression of ninein was significantly reduced in the GFP-electroporated area; however, the radial glial morphology of the APs was maintained, and the number of centrosomes remained unchanged at the ventricular zone surface of the *ninein* knockdown APs (Fig. 2B). Next, we evaluated the level of cell death using active caspase3 as a marker of apoptosis in our *ninein* siRNA experiments. No significant effects were observed in the control or the *ninein* knockdown neocortex (supplementary material Fig. S1).

Based on the siRNA conditions described above, we examined the distribution of M-phase (GFP^+^PH3^+^) and S-phase (GFP^+^BrdU^+^) cells in the neocortex of the control and *ninein* knockdown. In the control condition, approximately half of the mitotic cells were located at the ventricular surface, while the other half of the cells were divided in the SVZ as IPs (Fig. 2C). In the *ninein* knockdown, approximately half of the PH3^+^ M-phase cells were located near the VZ, although more mitotic cells were located at the non-ventricular surface and the apical region of the SVZ compared with the control (Fig. 2C). In quantitative analyses of the distribution of PH3^+^ cells, a large number of dividing cells were identified at the ventricular surface in the control slices (49.6%, *n* = 5), while the remaining dividing cells were found on the basal side of the VZ and SVZ (Fig. 2D, solid line). In contrast, in the *ninein* knockdown neocortex, the quantity of dividing cells in the basal side of the VZ and the apical side of the SVZ was greater (12.9±9.7% in 50–100 µm from the ventricular surface; 21.7±13.5% in 100–150 µm, *n* = 6, Student's *t-test P*<0.05) than the quantity observed in the control (0±0% in 50–100 µm; 6.3±9.8% in 100–150 µm, *n* = 5). Consistent with these data, the number of dividing cells in the *ninein* knockdown decreased (10.8±4.7% in 150–200 µm, *n* = 6, *P*<0.005; 1.6±3.8% in 200–250 µm, *n* = 6, *P*<0.05) at the basal side of the SVZ (Fig. 2D, dotted line) compared to the control neocortex (23.9±6.5% in 150–200 µm; 20.9±16.3% in 200–250 µm, *n* = 5). We also found that the mitotic indices of the VZ electroporated with *ninein* 947 si (2.3±0.2%, *n* = 18, *P*<0.01) or *ninein* 2580 si (2.7±0.7%, *n* = 18, *P*<0.005) were significantly less than those of the controls (3.8±0.8%, *n* = 7) (Fig. 2E), suggesting that the downregulation of ninein causes mis-localization of M-phase cells and decreases AP proliferation.

To further elucidate the function of ninein, we performed rescue experiments using an siRNA-resistant ninein-overexpression construct. As expected, ectopic mitosis was markedly reduced in the basal side of the VZ and the apical side of the SVZ within the wild-type cortex electroporated with both *ninein* 947 si and the overexpression construct (5.7±4.0% in 50–100 µm; 7.1±3.7% in 200–250 µm, *n* = 3, *P*<0.05) compared to that in the cortex electroporated only with *ninein* 947 si (12.9±9.7% in 50–100 µm; 1.6±3.8 in 200–250 µm, *n* = 6) (Fig. 2D). These partial rescue data suggest that the position of mitosis in the developing cortex may be dependent on the function of ninein.

Regarding the position of S-phase cells, BrdU-incorporated cells were located in the basal half of the VZ in the control neocortex, while those in the *ninein* knockdown neocortex were scattered within the VZ and SVZ (Fig. 2F). Among cells that were unlabeled with GFP, some BrdU^+^ cells were located in ectopic positions. Although we cannot exclude the possibility of non-cell autonomy effects of *ninein* knockdown, we assume that some siRNA was electroporated into cells without GFP because the molecular size of siRNA is much smaller than that of GFP plasmids. Quantitative analyses revealed that the number of BrdU^+^ cells at the apical side of the VZ transfected with *ninein* 947 si was significantly increased (29.2±13.6% in 0–50 µm, *n* = 6, *P*<0.05) when compared with the control (8.6±11.8% in 0–50 µm, *n* = 3) (Fig. 2G). The downregulation of ninein with either 947 si (10.5±1.3%, *n* = 18, *P*<0.005) or 2580 si (9.0±1.3%, *n* = 18, *P*<0.002) also led to markedly reduced BrdU incorporation compared with the control (15.8±2.6%, *n* = 5) (Fig. 2H). Moreover, the number of BrdU^+^ cells at the basal side of the VZ and the apical side of the SVZ was greatly reduced in samples transfected with ninein 947 si plus si-resistant ninein (20.3±10.2% in 0–50 µm, *n* = 3, *P*<0.05) compared with *ninein* knockdown (29.2±13.6%, *n* = 6); this finding demonstrates partial rescue (Fig. 2G). These results suggest that the mis-localization and decrease of both M- and S-phase cells in the neocortex observed in *ninein* knockdown may indicate abnormal INM during the S to M phase. Taken together, it is possible that ninein regulates at least the downward movement of the nucleus of APs during the S-G2-M phase.

Previous reports demonstrate that the orientation of the cleavage plane is an important factor influencing cell fate determination during cortical development (Chenn and McConnell, 1995; Haydar et al., 2003; Kosodo et al., 2004; Konno et al., 2008). To further characterize the extent to which *ninein* knockdown affects the cell fate determination of APs, we analyzed the mitotic orientation of APs at E17.5 in the neocortex that was electroporated at E15.5 (supplementary material Fig. S2A). There was no difference in the cleavage plane orientation at the apical division between cells transfected with *ninein* 947 si or the scrambled control siRNA (supplementary material Fig. S2B).

We next investigated whether overall neuronal production is affected by the removal of ninein by examining the cell cycle exit rate in the neocortex 24 hours after BrdU labeling at E16.5. The cell cycle exit rate was calculated as the number of Ki67^−^BrdU^+^ cells among the total number of BrdU^+^ cells. *Ninein* knockdown by 947 si led to a significantly increased (69.1±5.1%, *n* = 3, *P*<0.0005) cell cycle exit rate in the 947 si neocortex compared with that in the scrambled control (32.0±5.9%, *n* = 4) (supplementary material Fig. S2C,D). Therefore, ninein is required to maintain apical progenitors. The influence of *ninein* knockdown on cleavage plane orientation and cell cycle exit rates in our study in rats was similar to that previously reported in mice (Wang et al., 2009). Taken together, these data suggest that *ninein* knockdown causes a premature depletion of proliferating progenitors.

### The downregulation of ninein blocks INM in APs

**Fig. 3.**
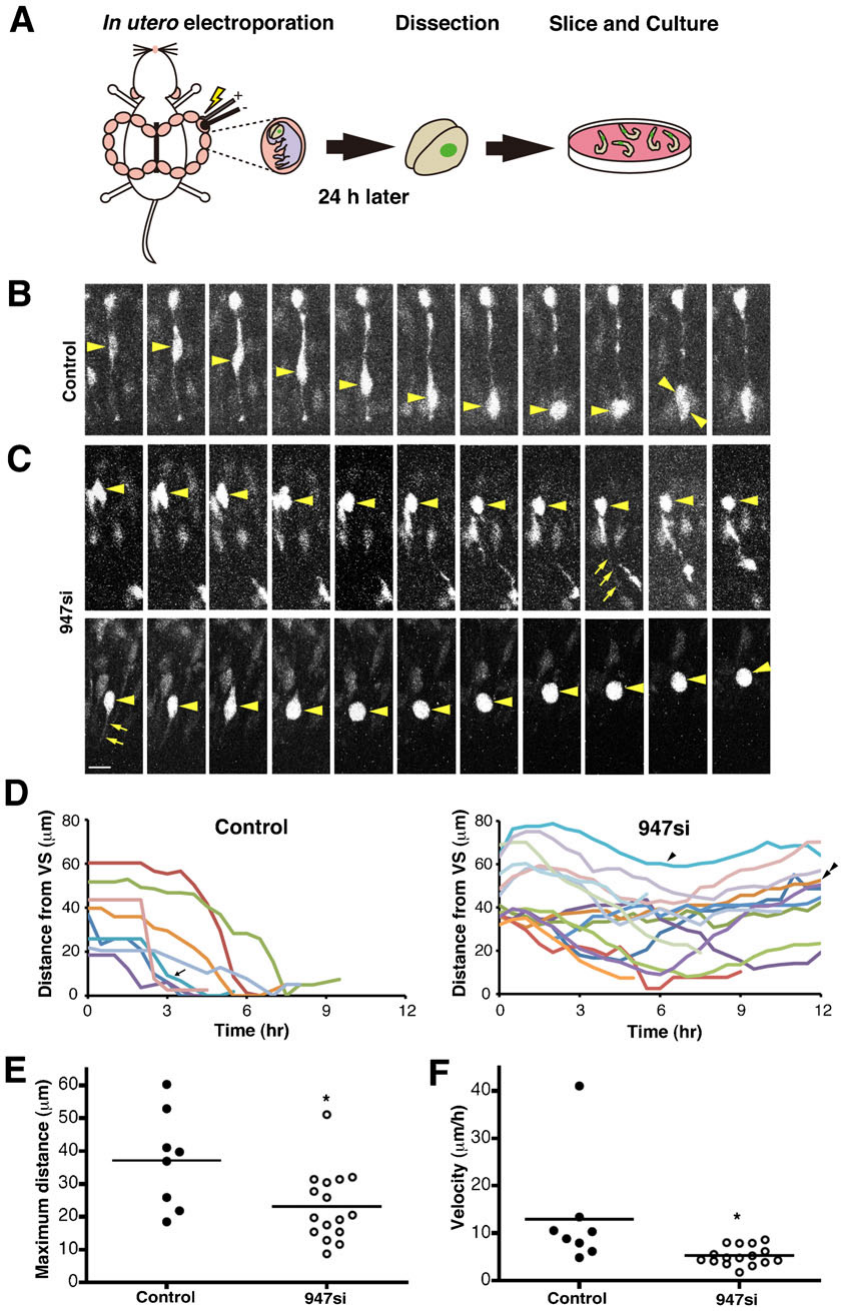
The loss of ninein causes abnormal interkinetic nuclear migration. (**A**) Schematic representation of *in utero* electroporation and slice culture. (**B**,**C**) Time-lapse fluorescent micrographs of RFP-expressing neuroepithelial cells with *ninein* control siRNA (B) and RFP-expressing neuroepithelial cells in the *ninein* knockdown by 947 si (C) (30 minutes intervals). Arrowheads and arrows indicate the position of the nucleus and apical process of a RFP-labeled neuroepithelial cell, respectively. (**D**) Tracings show the distance from the ventricular surface to nuclear position in control (*n* = 8, an arrow indicates the cell shown in B) and *ninein* knockdown (by 947 si) (*n* = 17, an arrowhead and a double arrowhead indicate the cells on the top and bottom panels in C) cells. (**E**) Maximum distances of nuclear migration before mitosis (23.15±2.67 µm, *n* = 16, *P<0.05). (**F**) Velocities of nuclear migration before mitosis (5.24±0.50 µm/hour, *n* = 16, *P<0.05). Scale bars: 10 µm in B,C.

The above data suggest that INM of APs is impaired in the *ninein* knockdown condition. Therefore, we attempted to observe the INM phenotype directly using time-lapsed imaging by confocal microscopy of acute slices after *in utero* electroporation (Fig. 3A). In the scrambled control siRNA-transfected cells, the nucleus migrated apically during the G2 phase and divided at the ventricular surface (8/8 cells) (Fig. 3B; supplementary material Movie 1). As a result, the nuclei transfected with the control si traversed long distances from the basal to the apical side during the imaging period (Fig. 3D); the maximum distance from the starting location was 37.13±5.21 µm (*n* = 8) (Fig. 3E), and the average velocity was 12.89±4.13 µm/hour (*n* = 8) (Fig. 3F). In contrast, nuclear migration toward the ventricular surface was severely impaired in cells transfected with *ninein* 947 si (Fig. 3C; supplementary material Movies 2, 3), although these *ninein* knockdown cells did exhibit radial glial morphology with apical processes (arrows in Fig. 3C). As expected, the nuclei of APs transfected with *ninein* 946 si did not reach the ventricular surface by wobbling. They travelled shorter distances (23.15±2.67 µm, *n* = 16, *P*<0.05) than the control APs (37.13±5.21 µm, *n* = 8, Fig. 3D,E) and at a slower velocity (5.24±0.50 µm/hour, *n* = 16, *P*<0.05) than the controls (12.89±4.13 µm/hour, *n* = 8) (Fig. 3F). Furthermore, cell divisions were detected only in 6/17 APs transfected with *ninein* siRNA during the time-lapsed observation period, as shown in Fig. 3C. These results indicate that *ninein* knockdown in APs leads to impaired nuclear migration during the S to M phase in INM.

Next, to further characterize whether the removal of ninein affects not only APs but also the production of IPs that are located in the SVZ and typically produce neurons after a limited number of cell divisions (Haubensak et al., 2004; Miyata et al., 2004; Noctor et al., 2004), we used the anti-Tbr2 antibody to detect IPs and examined the distribution and number of GFP^+^Tbr2^+^ cells. Tbr2^+^ cells were located more apically than in the control (supplementary material Fig. S3A,B), although the number of these cells was not changed between the control and *ninein* knockdown conditions (28.3% in control; 23.3% in 947 si; 21.7% in 2580 si) (supplementary material Fig. S3C). These data suggest that ninein may not contribute to the production of IPs.

### Ninein constitutes a molecular link between microtubules and the centrosome in APs

While the mechanism underlying regulation of INM by ninein in APs is unknown, one possibility is that it occurs by the connection between microtubules and the centrosome (Delgehyr et al., 2005). In several mammalian cell lines, ninein is a key molecule linking microtubule nucleation and anchoring at the centrosome. Ninein moves bi-directionally along microtubules and supports the connection between microtubules and adherens junctions (Bouckson-Castaing et al., 1996; Mogensen et al., 2000; Dammermann and Merdes, 2002; Delgehyr et al., 2005; Moss et al., 2007). We then analyzed whether the functions of ninein in the cell lines were conserved in the APs of the developing neocortex via microtubule-regrowth assays using primary AP cultures (Delgehyr et al., 2005; Krauss et al., 2008).

**Fig. 4.**
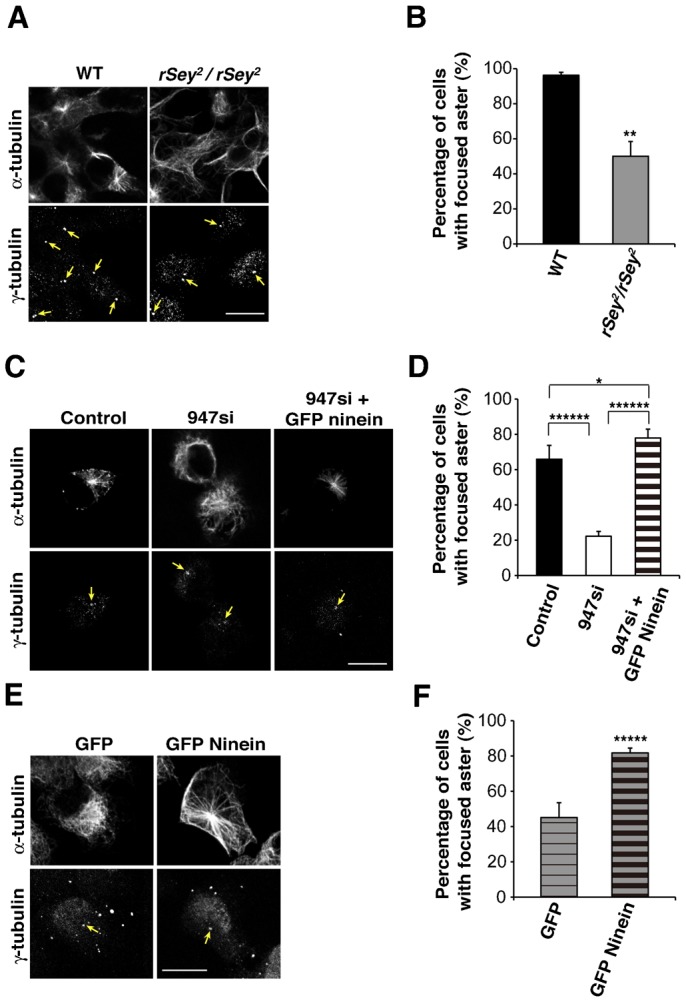
Ninein is required for microtubules aster organization in Aps. (**A**) Immunoreactivity of α-tubulin and γ-tubulin in APs taken from the wild-type (WT) or *Pax6* mutant rat 20 minutes after treatment with nocodazole in microtubule regrowth assay experiments. (**B**) Quantification of the WT (*n* = 134) and *Pax6* mutant (*n* = 136) APs with microtubule asters after a 20 minutes recovery period (**P<0.01). (**C**) Immunoreactivity of α-tubulin and γ-tubulin in WT APs transfected with control siRNA (left), *ninein*-947 si (middle) and *ninein*-947 si plus the GFP-ninein plasmid (right). Cells were treated with nocodazole for 24 hours post-transfection and recovered for 20 minutes. (**D**) Quantification of control siRNA and GFP-ninein-transfected WT APs with microtubule asters after a 20 minute-recovery period (88 (control), 90 (947 si) and 68 (947 si+GFP-ninein) cells were quantified, *P<0.05, ******P<5×10^−8^). (**E**) Immunoreactivity of α-tubulin and γ-tubulin in GFP- or GFP-ninein-transfected *Pax6* mutant APs. Cells were treated with nocodazole for 24 hours post-transfection and recovered for 20 minutes. (**F**) Quantification of GFP-and GFP-ninein-transfected *Pax6* mutant APs with focused microtubule asters after a 20 minute-recovery period (206 (GFP) and 296 (GFP-ninein) cells were quantified, *****P<5×10^−5^). Arrows indicate the position of centrosome. Scale bars: 10 µm.

First, we investigated whether the *Pax6* mutant APs showed impaired microtubule nucleation and anchoring. After 20 minutes of recovery from nocodazole treatment, wild-type APs exhibited well-organized radial arrays of cytoplasmic microtubules, whereas the formation of microtubule asters in the *Pax6* mutant APs was severely impaired (Fig. 4A). Quantitatively, the percentage of APs with microtubule asters was decreased after a 20 minutes of regrowth in *Pax6* mutant cells (50%, 68/136 cells, *P*<0.01) (gray bar in Fig. 4B) compared to the wild-type cells (96.3%, 129/134 cells) (black bar in Fig. 4B). These data suggest that microtubule nucleation is impaired in the Pax6 deficient condition.

We next examined whether *ninein* knockdown in APs taken from the wild-type neocortex induced similar effects. After a 20-minute recovery period, the radial arrays of cytoplasmic microtubules were well organized in 65.9% of the control siRNA-transfected cells (58/88 cells) (Fig. 4C; black bar in Fig. 4D), whereas the microtubule asters remained in only 22.2% of the cells transfected with *ninein* 947si (20/90 cells, *P*<5×10^−8^) (Fig. 4C; white bar in Fig. 4D). This reduction in cell number and in focused microtubule asters was restored by co-transfection with GFP-ninein (77.9%, 53/68 cells, *P*<5×10^−8^ vs *ninein* 947 si) and was slightly increased over the control (*P*<0.05 vs control siRNA) (Fig. 4C; striped bar in Fig. 4D).

Based on the above results, we tested whether ninein was able to rescue impaired microtubule nucleation derived from the *Pax6* mutant cortex in microtubule regrowth assays. After a 20-minute recovery period, 82.0% (242/296) of the GFP-ninein-overexpressing cells exhibited well-organized radial arrays of cytoplasmic microtubules (Fig. 4E,F), whereas microtubule asters were only observed in 46.2% (93/206) of the GFP-overexpressing cells (*P*<0.00005) (Fig. 4E,F). Therefore, the overexpression of ninein was able to rescue impaired aster formation.

Next, we tried to assess whether the connection between microtubules and centrosomes affects APs by removing ninein *in vivo*. Although this process was difficult to observe, cells transfected with control siRNA exhibited a certain amount of α-tubulin, a microtubule protein, in the apical process (arrowheads in supplementary material Fig. S4A, upper panel). In contrast, it seems that α-tubulin was absent in the apical processes of *ninein* 947 si-transfected cells (arrows in supplementary material Fig. S4A, lower panel). We carefully counted the number of cells with apical processes containing α-tubulin, and we found that the ratio of cells with α-tubulin-containing apical processes was significantly less in *ninein* 947 si-transfected cells (54.9±14.4%, *n* = 3, *P*<0.05) than in control cells (100±0%, *n* = 3) (supplementary material Fig. S4B). These data may imply that ninein is required for the connection between microtubules and the centrosome in APs.

**Fig. 5.**
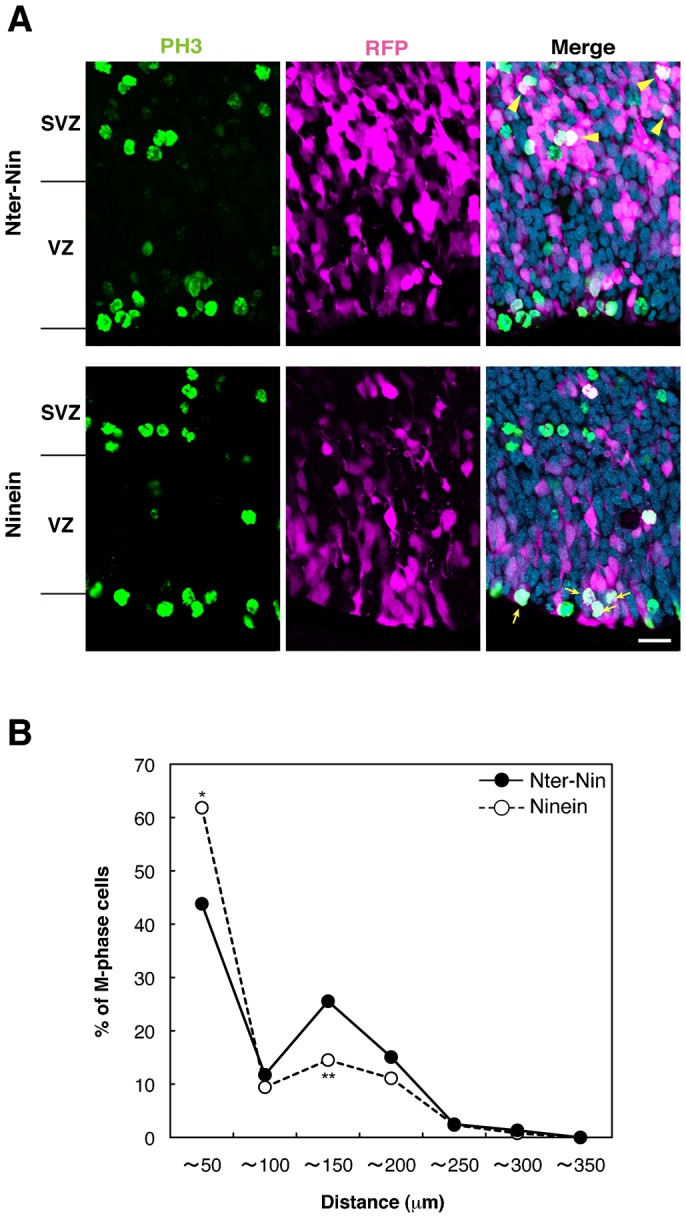
Ninein is required for maintaining the INM in the *Pax6* mutant. (**A**) Immunoreactivity of PH3 (green) and RFP (magenta) together with merged images of the cells transfected with the *ninein* N-terminal domain (Nter-Nin, upper panel) or *ninein* full length construct (Ninein, lower panels) in the *Pax6* mutant neocortex 48 hours after electroporation at E15.5. (**B**) Graphs showing the distribution of M-phase cells in samples transfected with *Nter-Nin* (dotted line) or *Ninein* (solid line) constructs. The number of PH3^+^GFP^+^ cells was calculated at 50 µm intervals from the apical surface up to 350 µm, and shown as a percentage of the labeled cells in each area against the total PH3^+^GFP^+^cells (*n* = 3–4, *P<0.05). Note that *ninein* overexpression rescued the ectopic position of mitotic cells in the *Pax6* mutant cortex (arrows), in contrast to the position of *Nter-Nin*-transfected mitotic cells (arrowheads). VZ, ventricular zone; SVZ, subventricular zone. Scale bar: 20 µm.

Finally, we performed rescue experiments *in vivo* by overexpressing *ninein* into *Pax6* mutant APs. As a control experiment, we overexpressed the construct containing only the *ninein* N-terminal domain (Fig. 5A, upper panel) because the ninein C-terminus is important for targeting the centrosome (Delgehyr et al., 2005). In quantitative analyses of the distribution of PH3^+^ cells, 43.8% of the dividing cells were identified at the ventricular surface of the *ninein* N-terminal domain-transfected cells, while the remaining dividing cells were found at the basal side of the VZ (25.6% in 100–150 µm, 4 independent experiments, upper panel in Fig. 5A and solid line in Fig. 5B). These features were similar to those in the non-transfected *Pax6* mutant neocortex (see figure 1 in Tamai et al. (Tamai et al., 2007)). In contrast, *ninein* full length-transfected neocortical cells (lower panel in Fig. 5A and dotted line in Fig. 5B) exhibited increases in the number of dividing cells in the apical side of the VZ and decreases in the basal side (i.e. 61.8% in 0–50 µm from the ventricular surface; 14.5% in 100–150 µm, *n* = 3, Student's *t-test P*<0.05). These cells were considered to be a recovered phenotype similar to those in the non-transfected wild-type neocortex (see figure 1 in Tamai et al. (Tamai et al., 2007)). Taken together, all these data consistently support the idea that ninein is required to connect microtubules and the centrosome in APs.

### Downregulation of ninein enlarged the endfoot area of APs

**Fig. 6.**
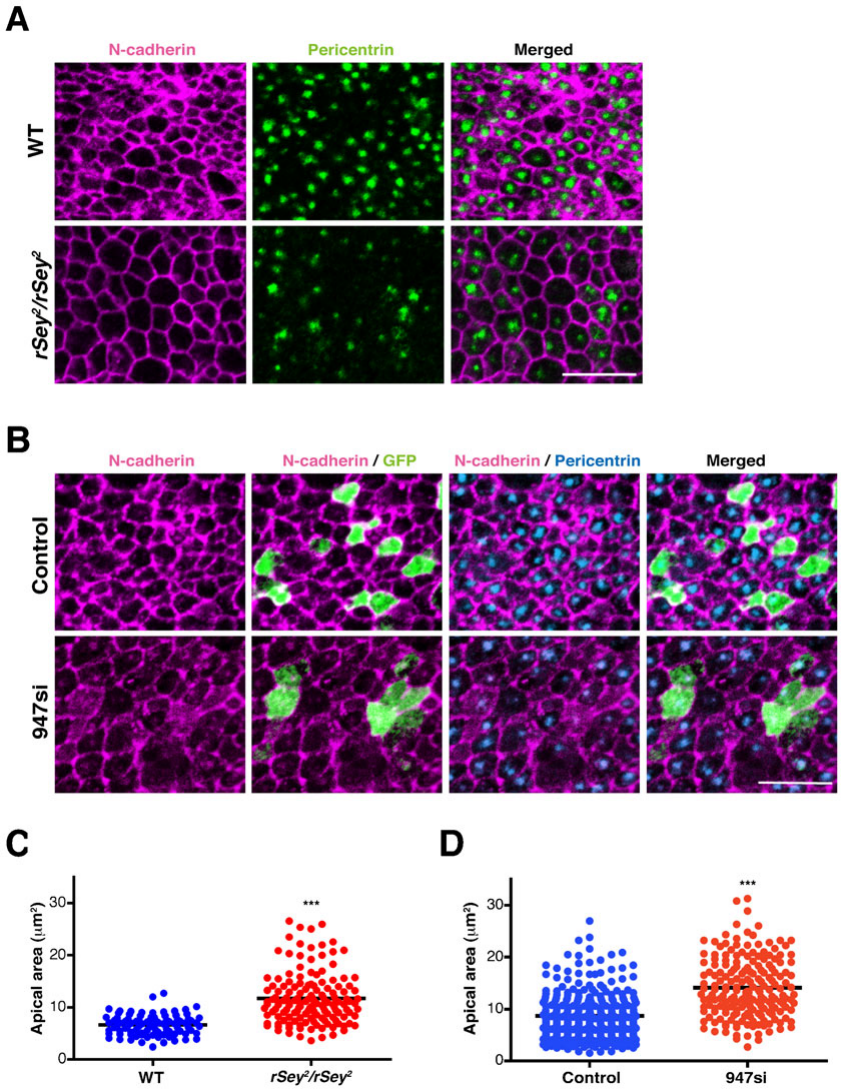
Ninein is required for maintaining the size of endfoot of the Aps. (**A**) *En face* view of immunoreactivity of N-cadherin (magenta) and pericentrin (green) in the wild-type (WT) and *Pax6* mutant neocortex at E17.5. (**B**) *En face* view of immunoreactivity of N-cadherin (magenta), GFP (green) and pericentrin (blue) in the control and *ninein* siRNA (947 si)-transfected APs 48 hours after electroporation at E15.5. (**C**) Graphs showing the average endfoot area of APs in the WT (*n* = 92, blue) and *Pax6* mutant neocortex (*n* = 146, red, ***P<0.0001). (**D**) Graphs showing the average endfoot area of APs transfected with control (*n* = 464, blue) and *ninein* siRNA (*n* = 191, red, ***P<0.0001). Scale bars: 10 µm.

During the course of our analyses of phenotypes of the *Pax6* mutant neocortex, we noted enlarged endfoot areas that were surrounded by N-cadherin-positive junctional meshwork (Nishizawa et al., 2007; Minobe et al., 2009) when assessing the *en face* view of the ventricular surface (Fig. 6A). A similar phenotype was observed by staining the tight junction protein ZO-1 (data not shown). Quantitatively, the average endfoot area of the E17.5 *Pax6* mutant neocortex (11.67±0.4024 µm^2^, *n* = 146, Student's *t-test P*<0.0001) was significantly larger than that of the wild-type (6.60±0.2006 µm^2^, *n* = 92) (Fig. 6A,C). Therefore, we further assessed whether knockdown of ninein also affected the size of the endfoot area of APs. The average endfoot area of the APs transfected with *ninein* siRNA (14.04±0.4100 µm^2^, *n* = 191, Student's *t-test P*<0.0001) was significantly larger than in the control APs (8.657±0.9625 µm^2^, *n* = 464) (Fig. 6B,D). It also seems that localization of N-cadherin was notably dispersed in *ninein* knockdown APs (Fig. 6B). This novel finding suggests that a Pax6-downstream molecule ninein may also regulate the size of the endfoot of APs.

## Discussion

Recent studies report that several centrosomal proteins are important for the regulation of INM (Xie et al., 2007; Zhang et al., 2009; Ge et al., 2010). In this study, we focused on the function of ninein, a centrosomal protein in APs that disappears in migrating neurons of the intermediate zone and is highly expressed in the cell soma and the dendrites of pyramidal neurons of the cortex (Ohama and Hayashi, 2009). In the *Pax6* mutant neocortex, which exhibits impaired INM, ninein expression is markedly reduced in the centrosome of the APs but remains detected in the cortical plate region. Consistently, the amount of *ninein* mRNA in the *Pax6* mutant neocortex decreased to half of that of the wild-type neocortex, although it was not completely lost at the transcript level. Considering the fact that Pax6 is specifically expressed in APs but not in pyramidal neurons (Osumi et al., 2008), ninein is considered a downstream molecule of Pax6. Searching the genomic sequence of rat ninein using the JASPAR CORE database, we found five prospective Pax6 binding sites (with matching scores 0.72 against Pax6 binding consensus sequence) on the 2 kb upstream to ninein ORF (902/915, 945/958, 1717/1730, 1806/1819, 1822/1835 bp). Therefore, the expression of the ninein gene might be directly regulated by Pax6 transcription factor in the developing rat brain. However, partial downregulation may imply that some indirect mechanisms may be involved in regulation of ninein expression and/or localization.

In a recent study, ninein expression was shown to be required to maintain self-renewal of APs in the mouse (Wang et al., 2009). In this study, we also observed reduced numbers of the S- and M-phase cells and increased cell cycle exit rates in the rat transfected with si-ninein, suggesting a conserved role of ninein in cell cycle exit. Although spindle position defects were observed in the E14.5 mouse cortex transfected with si-ninein (Asami et al., 2011), this phenotype was not reported in ninein knockdown observed at E16.5 by Wang et al. (Wang et al., 2009). Our results in the E17.5 rat (corresponding to the E15.5 mouse) were similar to that reported in Wang et al. (Wang et al., 2009). Therefore, we assume that the role of ninein may not primarily affect positioning of the spindle at mitosis. By combining our *Pax6* mutant rat phenotype analyses and functional assays *in vivo* and *in vitro*, we further revealed the importance of this ninein in INM. This feature occurred uniquely in APs. From our *in vitro* microtubule regrowth assays, we suspect that ninein is involved in INM via anchoring microtubules and the centrosome. Because ninein is a downstream molecule of Pax6 in the mouse (Asami et al., 2011), it is very likely that the abnormal nuclear migration during the S to G2 phase in the *Pax6* mutant cortex is due to reduced ninein expression (Tamai et al., 2007).

**Fig. 7.**
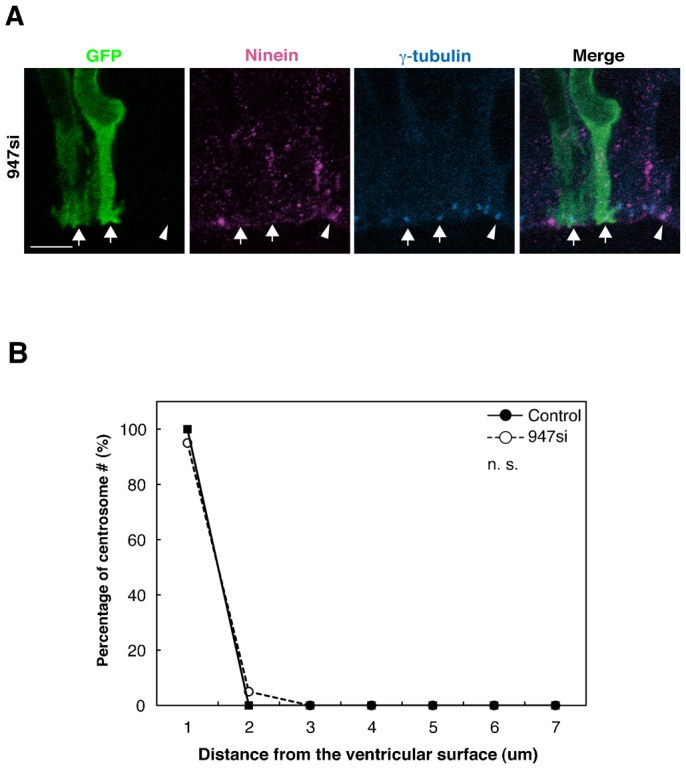
The localization of centrosome does not change in response to knockdown of ninein. (**A**) Immunoreactivity of GFP (green), ninein (magenta) and γ-tubulin (blue) in the neocortex of E17.5 *ninein* siRNA (947 si) transfected cells after 24 hours. Arrows indicate ninein^−^ γ-tubulin^+^ cells. Arrowhead indicates ninein^+^ γ-tubulin^+^ cells. (**B**) Quantitavive analysis of centrosomal position in the E17.5 neocortex transfected with *ninein* siRNA (947 si, dotted line, *n* = 14) or control siRNA (control si, solid line, *n* = 11). “n.s.” stands for not significant. Scale bar: 5 µm.

We previously reported unstable centrosome localization and impaired INM in the *Pax6* mutant APs (Tamai et al., 2007). However, as shown in Fig. 7A, most of the centrosomes remained at the apical surface region of the ninein-knockdown cortex. We have also included quantitative data on the centrosomal position in ninein knockdown and the control in Fig. 7B. In addition, the endfoot is clearly seen in the sample that was used for time-lapsed studies (Fig. 4). Therefore, we assume that ninein knockdown did not induce detachment of the endfoot in the radial/apical progenitors. Detachment in *rSey^2^/rSey^2^* could be related to diminished Fabp7, another Pax6 downstream molecule, in the developing rat cortex, although *Fabp7* does not seem to be under the control of Pax6 in the embryonic mouse cortex (Arai et al., 2005). Thus, some other molecule(s) must be generally responsible for maintaining the apical endfoot in the rodent cortex. In this regard, downregulation of another apical protein, δ-catenin, has been described using a transcriptome analysis of the cortex of *Pax6* mutant mouse (Duparc et al., 2006). We also identified decreased expression levels of δ-catenin and FEZ1 in our microarray analyses (Shinohara et al., manuscript in preparation). It would be interesting to determine the functions of these apical molecules to better understand the mechanism of INM and its related subcellular features.

In the *en face* view, we observed enlargement of the ventricular surface size of APs in the *Pax6* mutant cortical primordium. The phenotype looks similar to a *Pax6* mutant hindbrain that we observed previously (Takahashi and Osumi, 2011). Because knockdown of ninein increased the ventricular surface size of APs, the same molecular mechanism may act in the hindbrain neuroepithelial cells. In epithelial cell lines, ninein is reported to support the interaction between microtubules and adherens junctions (Moss et al., 2007). We also observed dispersed immunoreactivity of N-cadherin, a component of the adherens junction, in the *ninein* knockdown APs. Therefore, enlargement of the ventricular surface size of the APs may also result from decreased adhesive components at the apical area.

Many reports demonstrate that Pax6 is a pivotal player in both APs and adult neural stem/progenitor cells (Manuel and Price, 2005; Osumi et al., 2008). Pax6 is also expressed in the neural progenitors situated in the outer SVZ in the primate neocortex (Hansen et al., 2010). One of the Pax6 downstream molecules that we have identified in the rat is fatty acid binding protein 7 (FABP7/B-FABP/BLBP), which is widely used as a marker for neural stem cells (Arai et al., 2005). We have revealed that Fabp7 is essential for maintaining embryonic and adult hippocampal neural stem/progenitor cells (Arai et al., 2005; Watanabe et al., 2007; Matsumata et al., 2012). We have also reported that Pax6 regulates fucosyltransferase IX synthesizing Lewis X antigen, which is another marker for neural stem cells (Shimoda et al., 2002). Here, we identified ninein, a downstream molecule regulated by Pax6, that is a centrosomal protein crucial for INM regulation, which is a cellular feature of APs. We further revealed that a genetic program of Pax6-ninein functions regulate the apical surface size of APs. The outcome of enlarged apical sizes of APs is unknown. However, Pax6 is considered to simultaneously orchestrate various features of neural stem/progenitor cells via the regulation of different target genes.

## Supplementary Material

Supplementary Material
