# Supplementary material for: Ninein is essential for the maintenance of the cortical progenitor character by anchoring the centrosome to microtubules
Source: Biol Open. 2013 Jun 10;2(7):739–49. doi: 10.1242/bio.20135231 (PMC3711042; doi:10.1242/bio.20135231)
Supplement: Supplementary Material [file supp_2_7_739__index.html]

Ninein is essential for the maintenance of the cortical progenitor character by anchoring the centrosome to microtubules — Ninein is essential for the maintenance of the cortical progenitor character by anchoring the centrosome to microtubules — Supplementary Material 

# Ninein is essential for the maintenance of the cortical progenitor character by anchoring the centrosome to microtubules

## 

**Files in this Data Supplement:**

- Supplementary Material - Hiroshi Shinohara et al. doi: 10.1242/bio.20135231
- Movie 1 - **Movie 1. Interkinetic nuclear movement of a normal apical progenitor.** A representative fluorescent time-lapse observation (30 minutes intervals) of a cell transfected with RFP-construct and control siRNA at E16.5 showing rapid downward movement of the cell body during the G2 phase. An arrowhead indicates the position of the nucleus of the RFP-labeled apical progenitor.
- Movie 2 - **Movie 2. Interkinetic nuclear movement of an apical progenitor in *ninein* knockdown.** A representative fluorescent time-lapse observation (30 minutes intervals) of a cell transfected with RFP-construct and *ninein* siRNA (947 si) at E16.5 showing that the cell body stays nearly at the initial position. An arrowhead and arrows indicate the position of the nucleus and apical process, respectively, of the RFP-labeled apical progenitor.
- Movie 3 - **Movie 3. Another example of interkinetic nuclear movement of an apical progenitor in *ninein* knockdown.** A representative fluorescent time-lapse observation (30 minutes intervals) of a cell transfected with RFP-construct and *ninein* siRNA (947 si) at E16.5 showing that the cell body stays nearly at the initial position. An arrowhead and arrows indicate the position of the nucleus and apical process, respectively, of the RFP-labeled apical progenitor.
