# Supplementary material for: Ninein is essential for the maintenance of the cortical progenitor character by anchoring the centrosome to microtubules
Source: Biol Open. 2013 Jun 10;2(7):739–49. doi: 10.1242/bio.20135231 (PMC3711042; doi:10.1242/bio.20135231)
Supplement: Supplementary Material [file supp_bio.20135231_bio.20135231-s1.pdf]

Biology Open

Supplementary Material

Hiroshi Shinohara et al. doi: 10.1242/bio.20135231

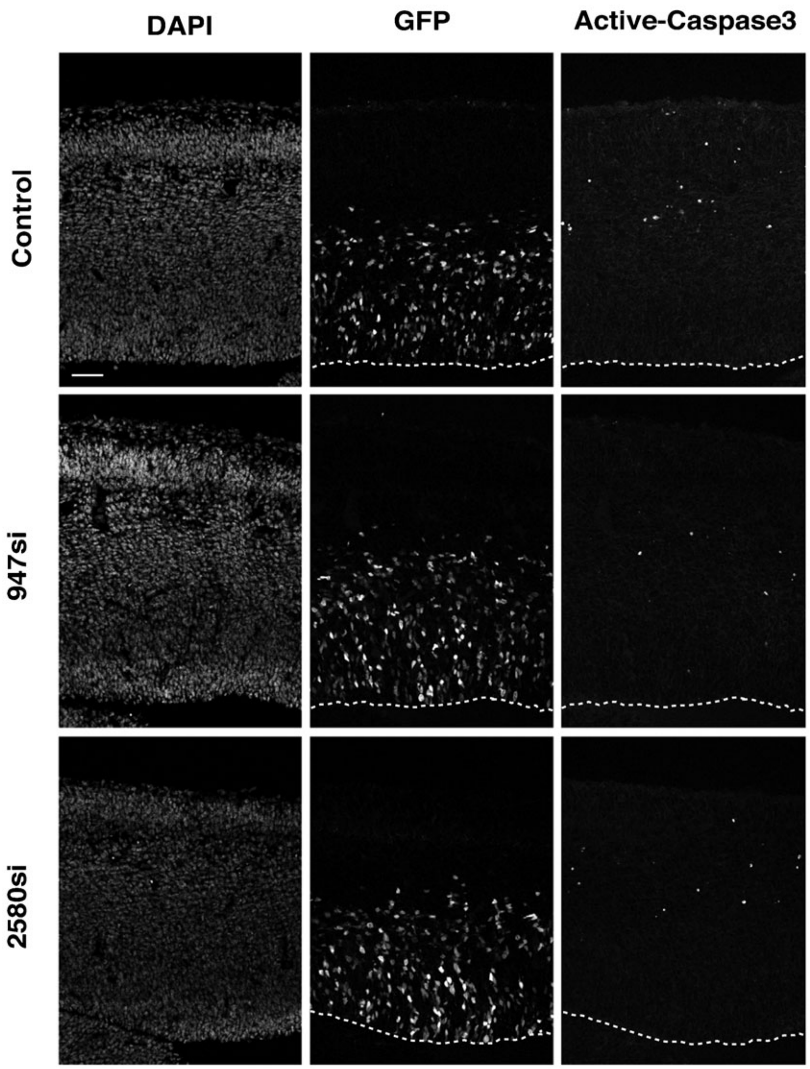

Fig. S1. Immunoreactivity of active-caspase 3 in *ninein* knockdown. Immunoreactivity of GFP (middle) and active-caspase3 (right) in the wild-type rat neocortical cells 24 hours after transfection with scramble control siRNA (top) or *ninein* siRNA (middle, 947 si; bottom, 2580 si) at E16.5. Broken lines indicate the ventricular surface. Scale bar: 20 µm.

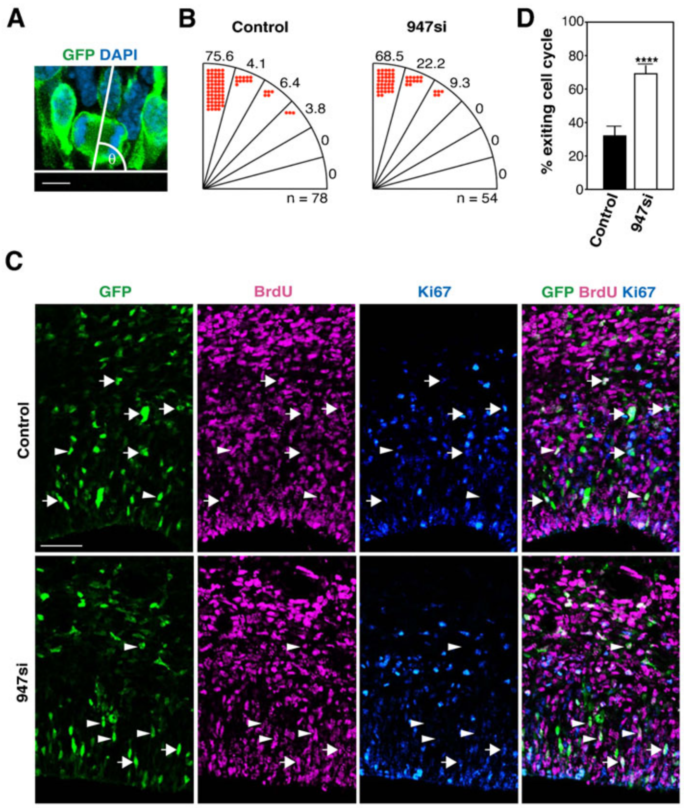

Fig. S2. Cleavage plane orientation and cell cycle exit rate in the *ninein* knockdown. (**A**) Schematic representation of the mitotic APs (from anaphase onward). The cleavage plane was defined as the orthogonal plane bisecting the line between the centers of each chromosome. The angle between the cleavage plane and the ventricular surface was measured. (**B**) Distribution of cleavage plane orientation at the apical divisions in the wild-type neocortical cells 48 hours after transfection of *ninein* siRNA (947 si and 2580 si)- or scramble control siRNA. (**C**) Immunoreactivity of GFP (green), BrdU (magenta) and Ki67 (blue) in the neocortex of control (top) and *ninein* siRNA (947 si) transfected (bottom) cells. Embryos were electroporated at E15.5, pulse-labeled with BrdU at E16.5, and sacrificed at E17.5. The cell cycle exit index was measured as the percentage of the GFP^{+} cells that exited the cell cycle (GFP^{+}BrdU^{+}Ki67^{-}) divided by the total GFP^{+}BrdU^{+} cells. Arrows indicate GFP^{+}BrdU^{+}Ki67^{+} cells. Arrowheads indicate GFP^{+}BrdU^{+}Ki67^{-} cells. (**D**) Quantification of cell cycle exit indices in the control siRNA and *ninein* siRNA (947 si) transfected cells (n=3–4, ****P<0.0005). Scale bars: 10 µm in A, 50 µm in C.

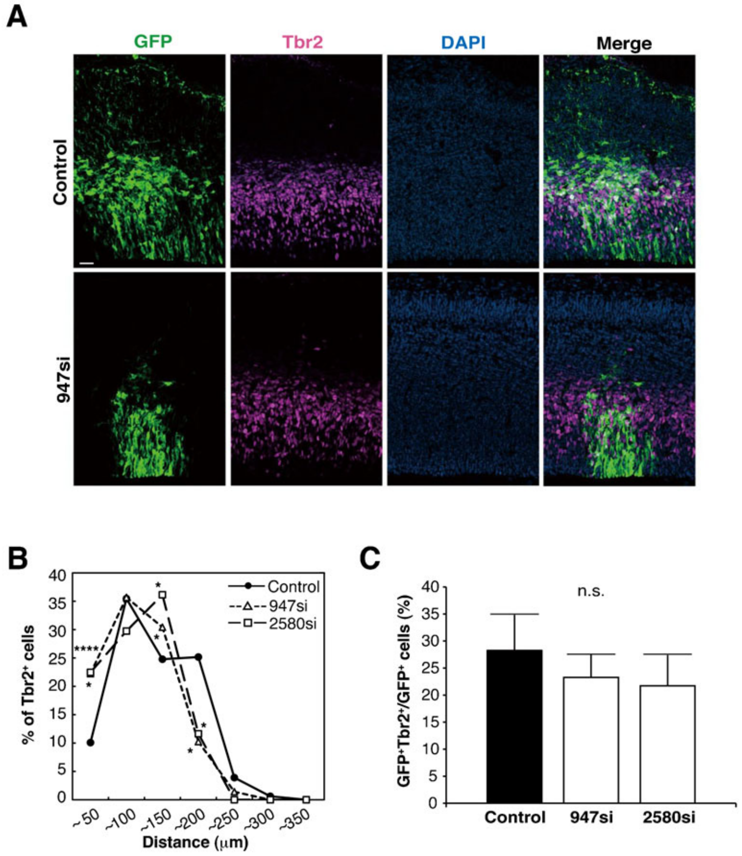

Fig. S3. Loss of ninein function randomizes the distribution of intermediate progenitor cells but does not affect their number. (**A**) Immunoreactivity of GFP (green), Tbr2 (magenta) in the wild-type neocortical cells 48 hours after transfection with control (top) or *ninein* siRNA (947 si) at E15.5. (**B**) The histogram shows the distribution of Tbr2^{+} IPs in *ninein* siRNA- and scramble control siRNA (solid line) transfected (947 si and 2580 si, dotted lines) cells. The number of Tbr2^{+}GFP^{+} cells was calculated from 0 µm from the apical surface to the basal area within 350 µm and quantified as a percentage of the labeled cells in each area against the total Tbr2^{+}GFP^{+}cells. Tbr2^{+}GFP^{+}cells of *ninein* knockdown embryos localize at the more apical side than in the control embryos (n=5, *P<0.05, ****P<0.0005). (**C**) The bar graph shows the percentage of Tbr2^{+}GFP^{+}cells to the total GFP^{+}cells in the SVZ of *ninein* siRNA (947 si and 2580 si, white bars)- and control siRNA (control si, black bar)-transfected neocortex (n=5). "n.s." stands for not significant. Scale bar: 20 µm.

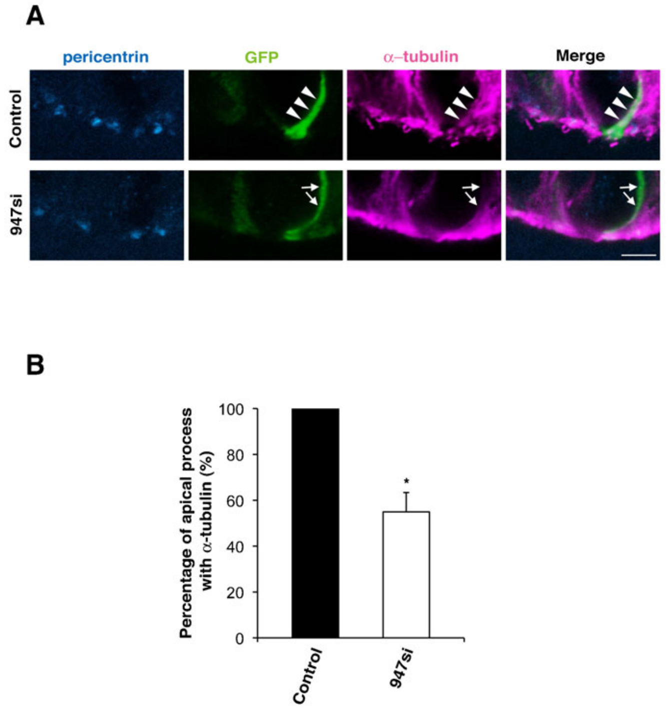

Fig. S4. Loss of ninein affects microtubule localization in the apical process of the AP. (**A**) Immunoreactivity of pericentrin (blue), GFP (green), α-tubulin (magenta), and merged images of control (upper panels) and *ninein* siRNA (947 si)-transfected (lower panels) cells 48 hours after electroporation at E15.5. In a 947 si-transfected cell, localization of α-tubulin is missing (arrows) compared with a control-transfected one (arrowheads). (**B**) Quantification of APs with apical processes showing localization of α-tubulin. Percentage of GFP^+ cells with *ninein*-947 si (n=30, right) is indicated against that of GFP^+ cells with control siRNA (n=29, left) (*$P<0.05$). Scale bar: 5 µm.

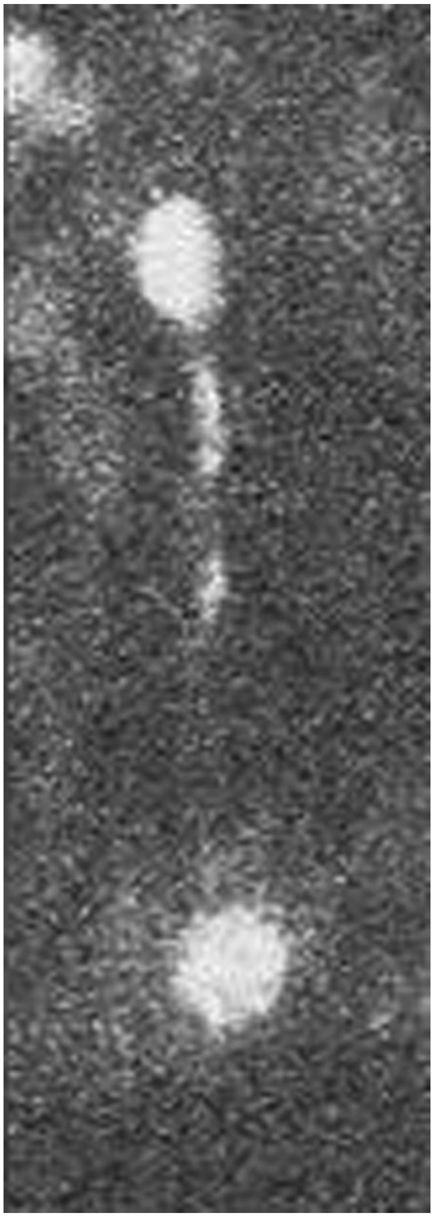

Movie 1. Interkinetic nuclear movement of a normal apical progenitor. A representative fluorescent time-lapse observation (30 minutes intervals) of a cell transfected with RFP-construct and control siRNA at E16.5 showing rapid downward movement of the cell body during the G2 phase. An arrowhead indicates the position of the nucleus of the RFP-labeled apical progenitor.

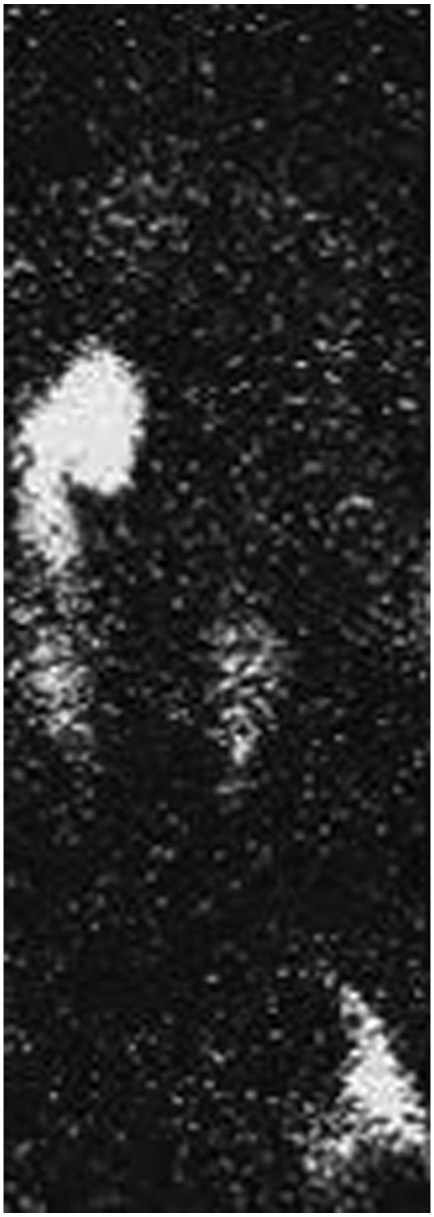

Movie 2. Interkinetic nuclear movement of an apical progenitor in *ninein* knockdown. A representative fluorescent time-lapse observation (30 minutes intervals) of a cell transfected with RFP-construct and *ninein* siRNA (947 si) at E16.5 showing that the cell body stays nearly at the initial position. An arrowhead and arrows indicate the position of the nucleus and apical process, respectively, of the RFP-labeled apical progenitor.

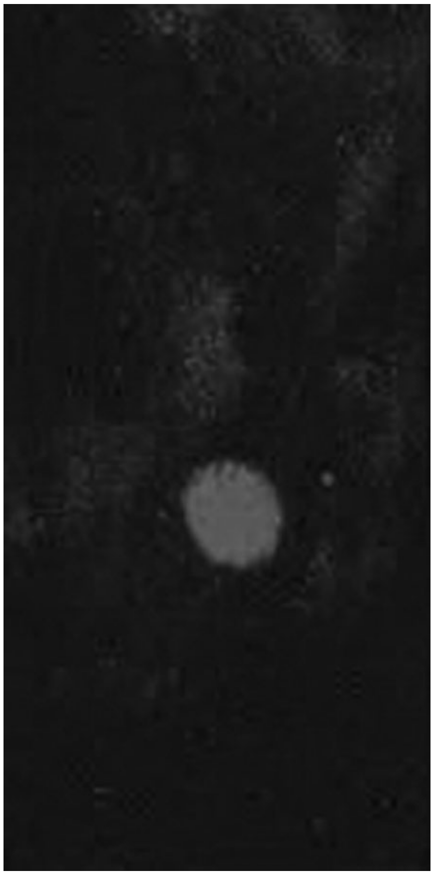

Movie 3. Another example of interkinetic nuclear movement of an apical progenitor in *ninein* knockdown. A representative fluorescent time-lapse observation (30 minutes intervals) of a cell transfected with RFP-construct and *ninein* siRNA (947 si) at E16.5 showing that the cell body stays nearly at the initial position. An arrowhead and arrows indicate the position of the nucleus and apical process, respectively, of the RFP-labeled apical progenitor.
